# Fresh transfer of an average quality slow growing day-3 embryo versus frozen transfer in a poor responder: a clinical management dilemma

**DOI:** 10.5935/1518-0557.20210095

**Published:** 2022

**Authors:** Garima Sachdeva, R Suchindra, Arveen Vohra, R Devi

**Affiliations:** 1 Milann, The Fertility Center, New Dheli, India

**Keywords:** *In vitro* fertilisation, embryo transfer, blastocyst, embryo, poor responder

## Abstract

A common conundrum faced by clinicians is whether to go for fresh transfer or culture the embryos for future frozen transfer in a case of slow-growing embryo. This case report describes a successful pregnancy with the fresh transfer of a single day 3- 6-cell grade B embryo in a patient with poor ovarian reserve. Although more research is needed in this context, the fresh transfer can be considered as a treatment option in patients with optimal endometrium and well-controlled progesterone levels with slow-growing embryos.

## INTRODUCTION

Embryo transfer can either be performed at a cleavage stage (day 3) or blastocyst stage (day 5). The blastocyst stage was once considered to be the best stage of embryos with the maximum clinical pregnancy rates. The logic behind this is that it improves the synchronicity between the embryo and the endometrium enabling embryo self-selection. However, even with the best culture media and condition, it is not necessary that all cleavage-stage embryos will convert to blastocyst. Actually, extended culture may sometimes harm the good quality embryos due to suboptimal culture conditions. According to the Cochrane review of 2016 (Glujovsky *et al*., 2016), there are no significant differences in the cumulative pregnancy rates between day-3 and day-5 transfers of fresh and frozen cycles.

Slower growing embryos are expected to have lower implantation and clinical pregnancy rates as compared to normal growing embryos (Shapiro *et al*., 2001). However, there is still a paucity of evidence regarding the better perspective, i.e., going for fresh transfer or continuing to culture and freezing the embryos for frozen transfer at a later date.

This report discusses the case of a patient in whom we transferred a slow growing average quality embryo in a fresh transfer cycle rather than culturing it further and freezing for transfer in subsequent cycles, whose outcome was a successful pregnancy.

## CASE REPORT

A 31-year-old woman visited our fertility clinic in March 2020 with a history of secondary infertility and a married life of six years. She had conceived naturally 3 years back, but since it was an unwanted pregnancy, medical termination was performed at 6 weeks of gestation. She had a regular, 28-day cycle. The patient did not have a significant medical or surgical history. She had no history of drinking or smoking. Her husband was 36 years old. He had a history of erectile dysfunction and hypertension, for which he was evaluated and given appropriate treatment. He did not drink or smoke. There was no other significant history.

Basic infertility evaluation showed her ovarian reserve was low, as indicated by an AMH (anti-Müllerian hormone) level of 0.1 ng/ml and an AFC (antral follicle count) of 3. Her baseline estradiol and FSH (follicle-stimulating hormone) levels were within normal limits - 28.5 pg/ml and 4.5 mIU/ml, respectively. Her hysterosalpingogram was suggestive of bilateral patent tubes. Semen analysis revealed normozoospermia. On further evaluation, she was diagnosed to have overt diabetes mellitus with a fasting blood sugar of 263 mg/dl and HbA1c of 12.7%). She was started on oral hypoglycaemic agents and her blood sugars were controlled over a period of three months.

**Figure 1:**
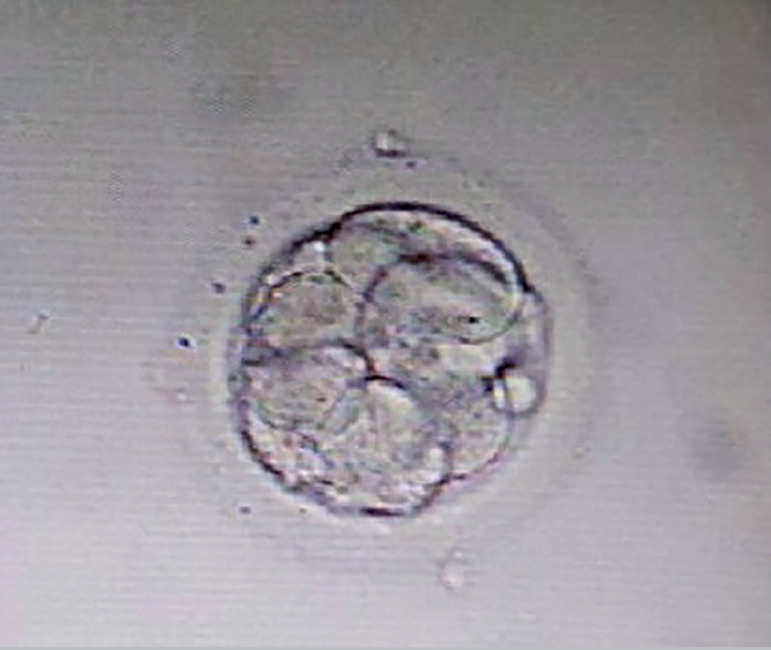
Day 3 slow growing embryo: 6-cell grade B.

The patient was counselled for the need of pooling IVF (*in-vitro* fertilization) in view of her poor ovarian reserve. The controlled ovarian stimulation cycle was started in June 2020 using the antagonist protocol. Only one dominant follicle (18.5 mm) developed after nine days of stimulation and a total of 2700 IU of gonadotropins (INJ GONAL-F (recombinant FSH)- 1875 IU + INJ HUMOG-HP (Highly purified HMG)- 825 IU). Ovulation was triggered with a dual trigger (Inj ovitrelle 250 mcg + Inj Decapeptyl 0.2 mg). Oocyte retrieval was performed 34.5 hours after trigger and one oocyte was retrieved and successfully fertilized. Progesterone level on the day of trigger was 0.1 ng/ml. Endometrial thickness on the day of pick-up was 10 mm. We planned for a fresh transfer on day 3 and started the patient on 50 mg intramuscular progesterone from the day of oocyte retrieval for 3 days. On day 3 (assessment was done 68 hours after insemination), we got a 6-cell grade B embryo (Alpha Scientists in Reproductive Medicine & ESHRE Special Interest Group of Embryology, 2011) (20% fragmentation) (Figure 1). The patient was counselled about the slow growing and average quality embryo. Fifteen days later, we got a positive beta- HCG report of 299.50 mIU/ml. The patient was followed with regular antenatal check-up and scans. At present, her pregnancy is progressing well with her blood sugars controlled on insulin. She is currently 26 weeks pregnant.

## DISCUSSION

The patient described in this case belonged to the Poseidon group 3 (Humaidan *et al*., 2016) and had a very low ovarian reserve (AMH- 0.1 ng/ml). The various options available for the treatment of such patients include oocyte retrieval followed by fresh transfer or pooling IVF cycle or counselling for donor oocyte IVF (Çelik et al., 2018). In this case, the patient was not in favour of donor oocytes. Also, other factors favouring optimal outcome with self- IVF included her age, previous history of natural conception, and normal FSH levels. Hence the decision of going ahead with self-oocyte was made.

However, despite stimulation with high doses of gonadotropin, we could get only one oocyte, which later fertilised. Here, fresh transfer was chosen over freezing and pooling IVF because of the cost factor involved in the vitrification of the embryos. Also, a study by Haas *et al*. (2019) demonstrated that when they transferred fresh slow growing embryos, the outcome was better as compared to culturing and freezing them and then thawing them later for a frozen transfer.

ACCUVIT (accumulation and vitrification) of embryos is an evidence-based effective treatment option in patients with very low ovarian reserves, like the one described in this case report (Cobo *et al*., 2012). However, the chances of a slow growing embryo converting to blastocyst on further culturing are not very good.

To conclude, if the endometrium is in optimal condition on the day of trigger and oocyte retrieval before ovum-pick up, with progesterone well controlled, one could consider the option of fresh transfer.

## CONCLUSION

Although more research is needed in this context, fresh transfer can be considered as a treatment option in patients with an optimal endometrium (pattern and thickness) and slow growing embryos.
